# Testing feedback message framing and comparators to address prescribing of high-risk medications in nursing homes: protocol for a pragmatic, factorial, cluster-randomized trial

**DOI:** 10.1186/s13012-017-0615-7

**Published:** 2017-07-14

**Authors:** Noah M. Ivers, Laura Desveaux, Justin Presseau, Catherine Reis, Holly O. Witteman, Monica K. Taljaard, Nicola McCleary, Kednapa Thavorn, Jeremy M. Grimshaw

**Affiliations:** 1 Women’s College Research Institute and Women’s College Hospital Institute for Health Systems Solutions and Virtual Care, Toronto, Canada; 2 Department of Family and Community Medicine, Women’s College Hospital, Toronto, Canada; 3 Institute for Clinical Evaluative Studies, Toronto, Canada; 4 Department of Family and Community Medicine and Institute of Health Policy, Management and Evaluation, University of Toronto, Toronto, Canada; 5 Clinical Epidemiology Program, Ottawa Hospital Research Institute, Ottawa, Canada; 6 School of Epidemiology, Public Health and Preventive Medicine, University of Ottawa, Ottawa, Canada; 7 Institut de recherche de l’hôpital Montfort, Ottawa, Canada; 8 Department of Family and Emergency Medicine, Laval University, Québec, Canada; 9 Office of Education and Continuing Professional Development, Laval University, Québec, Canada; 10 Laval University Research Institute for Primary Care and Health Services, Québec, Canada; 11 Population Health and Optimal Health Practices, CHU de Québec, Québec, Canada; 12 Department of Medicine, University of Ottawa, Ottawa, Canada

**Keywords:** Audit and feedback, High-risk prescribing, Nursing homes, Randomized trial

## Abstract

**Background:**

Audit and feedback (AF) interventions that leverage routine administrative data offer a scalable and relatively low-cost method to improve processes of care. AF interventions are usually designed to highlight discrepancies between desired and actual performance and to encourage recipients to act to address such discrepancies. Comparing to a regional average is a common approach, but more recipients would have a discrepancy if compared to a higher-than-average level of performance. In addition, how recipients perceive and respond to discrepancies may depend on how the feedback itself is framed. We aim to evaluate the effectiveness of different comparators and framing in feedback on high-risk prescribing in nursing homes.

**Methods:**

This is a pragmatic, 2 × 2 factorial, cluster-randomized controlled trial testing variations in the comparator and framing on the effectiveness of quarterly AF in changing high-risk prescribing in nursing homes in Ontario, Canada. We grouped homes that share physicians into clusters and randomized these clusters into the four experimental conditions. Outcomes will be assessed after 6 months; all primary analyses will be by intention-to-treat. The primary outcome (monthly number of high-risk medications received by each patient) will be analysed using a general linear mixed effects regression model. We will present both four-arm and factorial analyses. With 160 clusters and an average of 350 beds per cluster, assuming no interaction and similar effects for each intervention, we anticipate 90% power to detect an absolute mean difference of 0.3 high-risk medications prescribed. A mixed-methods process evaluation will explore potential mechanisms underlying the observed effects, exploring targeted constructs including intention, self-efficacy, outcome expectations, descriptive norms, and goal prioritization. An economic analysis will examine cost-effectiveness analysis from the perspective of the publicly funded health care system.

**Discussion:**

This protocol describes the rationale and methodology of a trial testing manipulations of theory-informed components of an audit and feedback intervention to determine how to improve an existing intervention and provide generalizable insights for implementation science.

**Trial registration:**

NCT02979964

**Electronic supplementary material:**

The online version of this article (doi:10.1186/s13012-017-0615-7) contains supplementary material, which is available to authorized users.

## Background

There is evidence that how audit and feedback (AF) is designed and delivered may impact the effectiveness of the intervention [1]. However, little attention has been paid towards identifying the best way to design AF to encourage changes in clinical practice, in order to resolve discrepancies between actual and desired performance [1]. Brehaut and colleagues identified 15 suggestions for improving the effectiveness of AF but highlighted numerous areas requiring further empirical research to address outstanding uncertainty [2].

One area of uncertainty relates to the choice of the comparator that reflects desirable practice in a feedback report. Most often, the recipients’ performance is compared against the average performance achieved by those practicing in the region. However, a higher benchmark more closely representing desired care would more clearly demonstrate the discrepancy between actual and desired performance for the majority of feedback recipients. A large randomized trial demonstrated greater improvements when feedback recipients were compared to an ‘achievable benchmark’, operationalized as the median score achieved by the top 10% of peers [3], versus those compared to the median [4]. To our knowledge, as is the case with many implementation science results [5], this has never been replicated.

Another area of uncertainty relates to the ‘framing’ of feedback content [6]. For a given quality indicator, it is possible to describe performance in multiple ways. For instance, a metric may be described as the proportion of patients for whom a desirable or guideline-concordant task was achieved (e.g. patients in whom high-risk medications were avoided). Alternatively, it may be described as the proportion of patients receiving care that is generally undesirable or not concordant with guidelines (e.g. patients inappropriately prescribed a high-risk medication). To our knowledge, despite extensive literature about how framing influences health-related decisions and behaviour in other contexts [7–12], the impact of different approaches to framing performance feedback provided to clinicians has never been tested.

We have previously argued that the science of AF is ‘stagnant’ [13] and that further trials testing AF against a usual care control group will not add new knowledge to the field [1]. Further, we have proposed that implementation scientists can partner with health systems or organizations already conducting interventions such as AF to create ‘implementation science laboratories’ in which various approaches to optimize such interventions can be tested [14]. In the present article, we describe a trial testing different AF designs, embedded within an implementation science laboratory.

## Methods

This is a pragmatic, 2 × 2 factorial, cluster-randomized trial testing variations in the comparator and in framing on the effectiveness of AF to change prescribing, in the context of an existing province-wide AF intervention. An embedded mixed-methods process evaluation will explore potential mechanisms underlying the observed effects. The protocol received ethics approval from the Research Ethics Boards at the University of Toronto and Women’s College Hospital. The Ottawa Health Science Network Research Ethics Board approved the process evaluation and the economic evaluation. The trial is registered on ClinicalTrials.gov (NLM identifier: NCT02979964).

### Setting

In the province of Ontario, Canada, medically necessary services, including a broad formulary of prescription medications for those living in nursing homes, are covered under the government-run, publicly funded health plan. Health Quality Ontario (HQO) is the provincial advisor on quality in healthcare and, as such, supports ways to improve healthcare quality through quality improvement initiatives. One such initiative is HQO’s ‘Practice Reports’, whereby confidential, aggregate feedback is offered to physicians across the province about their own practice. The reports for physicians working in nursing homes are known as the Long-Term Care Practice Reports (http://www.hqontario.ca/Quality-Improvement/Practice-Reports/Long-Term-Care). The HQO reports are generated by a multidisciplinary team, including clinicians, epidemiologists, and quality improvement experts, supported by input from front-line clinicians, health services researchers, sector organizations and associations, and policy makers. HQO relies on best evidence, advice from the groups listed above, and established methods (e.g. modified Delphi process) to select and develop indicators, and generate suggested action plans to support quality improvement for the Report.

In 2015, we developed an implementation science laboratory to support the optimization of HQO’s AF initiatives. This involves a concerted effort between implementation scientists and HQO to conduct applied research that will optimize the impact of HQO’s ongoing initiatives, while simultaneously contributing novel, generalizable findings that add to the scientific literature [14].

### Intervention design: long-term care practice reports

Details on the history of the long-term care practice reports and its re-design in preparation for this study can be found in Additional file 1.

Two members of the team (LD and JP) reviewed the content of the re-designed report to identify evidence-based behaviour change techniques (BCTs) [15] that were likely to contribute to behaviour change (i.e. the ‘active content’). This process was undertaken to ensure a standardized description of the report content with the goal of contributing to the broader understanding of how to design effective AF interventions [1]. The re-designed reports included a total of five BCTs that were operationalized throughout (summarized in Table 1).

**Table 1:** Behaviour change techniques included in re-designed reports across all trial arms

Behaviour change technique	Definition	Example of operationalization
Feedback on behaviour	Monitor and provide informative or evaluative feedback on performance of the behaviour (e.g. form, frequency, duration, intensity)	*7/72 of my residents were prescribed a benzodiazepine*
Social comparison AND Discrepancy between goal and behaviour	Draw attention to others’ performance to allow comparison with own performance AND Draw attention to discrepancies between a person’s current behaviour (in terms of the form, frequency, duration, or intensity of that behaviour) and the person’s previously set outcome goals, behavioural goals or action plans (goes beyond self-monitoring of behaviour)	*7 additional residents in my practice may be at increased risk associated with benzodiazepines (compared to Ontario long-term care physicians with lower prescribing rates)*
Information about health consequences	Provide information (e.g. written, verbal, visual) about health consequences of performing the behaviour	*How many of my residents are exposed to risks (e.g. falls) related to benzodiazepines?*
Problem solving	Analyse, or prompt the person to analyse, factors influencing the behaviour and generate or select strategies that include overcoming barriers and/or increasing facilitators	*Change ideas, worksheets and resources regarding how to implement best practices for prescribing*

Definitions taken directly from Michie et. al. [15]

### Program theory and logic model

**Fig. 1:**
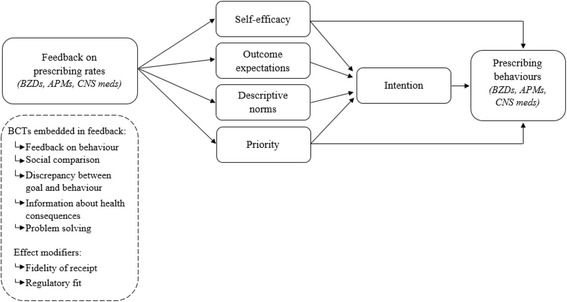
Theory-informed logic model. *APM* antipsychotic medication, *BCT* behaviour change technique, *BZD* benzodiazepine, *CNS* central nervous system

We developed the program theory [16] for the AF intervention in a manner consistent with the hypotheses proposed, and the theory and evidence underlying these (refer to Additional file 2). We defined the intervention according to the BCTs used to target hypothesized constructs, using terminology described by the Behaviour Change Technique Taxonomy v1 [15] and informed by techniques described by goal-setting theory [17], Social Cognitive Theory [18, 19], and nascent theory characterizing the role of competing priorities in healthcare professional behaviour change [20–22]. We then summarized the program theory as a path analytical model to inform the mechanistic theory-based process evaluation (Fig. 1).

### Experimental conditions

All participants will be sent a version of the HQO Practice Reports (Additional file 3) which include the BCTs outlined above. For the study, we manipulated two features of the feedback which may be thought of as experimental factors: (1) the comparator (average vs top quartile) and (2) the framing of the feedback content (patients at risk vs patients safe from risk). Thus, we created four versions of the HQO Practice Reports (Table 2).

**Table 2:** Factorial 2 × 2 diagram of trial arms

	Factor 1: Comparator [Top quartile/average]	
Factor 2: framing [Benefit framing/risk framing]	75th percentile/benefit framing	Average/benefit framing
	25th percentile/risk framing	Average/risk framing

### Factor 1: benchmark/comparator in feedback reports

Participants’ prescribing rates will be compared to either the provincial average or top performing peers (top quartile). Prior to this trial, HQO practice profiles compared performance of the recipient to provincial averages on a summary page and both provincial and regional averages on the detailed indicator pages. One of the recently published suggestions for practice feedback design is to use ‘a single comparator that effectively communicates the key message’ [2]. Comparing recipients to the top 10% of their peers is more likely to achieve this aim than comparing to the average score of all their peers [4]. However, HQO felt that a benchmark of the top 10% may risk unintended discontinuation of medications that may be actually be appropriate, rather than a careful review of medication use. The top quartile was decided to be acceptable for the purposes of the trial while maintaining the essential needs of the HQO program that avoids unnecessary harms to residents in LTC.

### Factor 2: framing of performance in feedback reports

Factor 2 tests the framing of the feedback reports to be either ‘risk-framed’ (e.g. emphasizing patients at risk of harms) or ‘benefit-framed’ (e.g. emphasizing patients *safe* from risk of harms) without changing the underlying data. Decision-making is affected by the way a problem (or in this case, feedback) is framed [11, 23]. Framing has been shown to affect clinical decision-making of physicians in prior studies [24], with the perceived level of risk involved in the decision posited as a mediating factor [25]. Prospect theory describes how people may differentially weigh the potential gains and potential losses, emphasizing the importance of risk aversion in decision-making [26].

We operationalized *risk framing* by providing feedback on the proportion of patients receiving high-risk medications and describing this as patients *at risk of harm* from those medications. This framing was designed to be presented in two ways: visually using the line graph with percentage of patients at risk on the *y*-axis and red colour emphasis in the graph and in text form with the statement ‘Nn additional/fewer resident(s) in my practice may be/are at increased risk associated with benzodiazepines’ (see Additional file 4). We operationalized *benefit framing* by providing feedback on the (1-X)% of patients in whom high-risk medications were avoided and described this as patients *safe from the risks of harm* associated with those medications. This framing was designed to be presented in two ways: using a line graph with percentage of patients safe from risk on the *y*-axis and green colour emphasis in the graph and in text form with the summary statement ‘No additional/fewer resident(s) in my practice may be/are safe from risks associated with benzodiazepines’ (see Additional file 4).

We refined the language for risk framing and the associated colour changes iteratively through a user-testing process (Additional file 1) to ensure that the emotional responses invoked would encourage action rather than distract from the task of improvement [6].

### Eligibility

Physicians who (1) voluntarily signed up to receive an HQO Practice Report prior to randomization and (2) consistently have >5 residents that they care for in the nursing home setting (to allow for adequate data capture) will be included in the trial. We will include data from nursing home residents if the residents’ most responsible physician is eligible.

### Allocation

Cluster randomization is necessary because the intervention is delivered to physicians who may work collaboratively with other health care professionals. To prevent contamination that could occur when physicians work in multiple nursing homes, we have grouped homes that share physicians into clusters. Thus, the unit of randomization will be groups of one or more nursing homes sharing physicians. An independent statistician will randomize these clusters independently by the two factors (resulting in four experimental conditions), stratifying by total number of beds in the cluster [27], using a randomly permuted block design of length four. Physicians who sign up for the report after randomization and who belong to a cluster already assigned will be provided the same allocation as others in their group to avoid contamination.

### Blinding

Participants in this study (i.e. physicians) are not explicitly blinded, but the risks in this instance are felt to be minimal, as they will not be aware of the variations being tested nor the outcome measures and analytical approach. Further, the trial occurs in the context of a newly designed report that incorporates new quality indicators. The analysts will be blind to allocation status.

### Data collection

Quality indicators collected for use in the feedback reports and related clinical measures are derived from routinely collected administrative data, linked using unique encoded identifiers at the Institute for Clinical Evaluative Sciences (ICES).

Data will be compiled from the following databases: (1) the Ontario Drug Benefits (ODB) database, covering nearly all prescriptions in nursing homes; (2) the Canadian Institute for Health Information (CIHI) databases covering all inpatient hospitalizations and emergency department visits; (3) the Ontario Health Insurance Plan (OHIP) database, covering physician billings; (4) the Registered Persons Database (RPDB) covering demographic information including date of death; and (5) the Continuing Care Reporting System (CCRS) database for clinical and demographic information on nursing home residents collected using the Resident Assessment Instrument (RAI). The RAI is legislatively required for each nursing home resident in the province within 2 weeks of admission date and then every 92 days, or any other time when the clinical condition of a resident considerably changes. The RAI captures a range of data such as functional and cognitive performance scales and can be used to determine date of admission and discharge from each nursing home [28].

HQO will track the unique College of Physician and Surgeons of Ontario (CPSO) numbers for those physicians who signed up for the reports. This will be encrypted and linked to the ICES Physician Database to assess physician characteristics, including age, sex, and whether they graduated from Canada or an international school. For each physician who signs up for the Practice Reports, HQO will also keep a file to track the number of times they downloaded the report at each quarterly release. This data will be confidentially shared with ICES analysts (keeping investigators blind), and the coded data will be used to conduct a sensitivity analysis to examine how intensity of exposure relates to effect size.

### Outcomes

All primary and secondary outcomes of interest are summarized in Table 3 and are defined at the individual resident level. The primary outcome is the monthly number of CNS-active medications per resident, as determined from the ODB database. ‘CNS-active’ medications will be defined in a manner consistent with the HQO quality indicator used in the Practice Reports, covering the medication classes of antipsychotics, opioids, benzodiazepines, and antidepressants (including tricyclic antidepressants and trazodone). This was chosen as a way of capturing any change to prescribing that was directly influenced by the measures provided in the Practice Reports. Secondary prescribing outcomes will include additional measures of prescribing that might be directly influenced by the Practice Reports: proportion of days supplied benzodiazepines, any prescription of benzodiazepines, mean daily benzodiazepine dose, dose equivalent of benzodiazepine dispensed, proportion of days supplied antipsychotics, any prescription of antipsychotics, mean daily antipsychotic dose, dose equivalent of antipsychotic dispensed, and presence of three or more CNS-active medications. We will also assess statin prescriptions as a non-targeted control or ‘tracer outcome’ [29]. These are not expected to be affected by the intervention and will be used to identify secular trends in prescribing in the homes.

**Table 3:** Primary and secondary outcomes—all defined at the level of the individual resident

Outcomes	Scale	Number of repeated measurements
Primary outcome measure		
Number of CNS-active medications (antipsychotics, opioids, benzodiazepines, or antidepressants (including tricyclic antidepressants (TCAs) and trazodone))	Continuous	Monthly
Secondary outcome measures		
Benzodiazepine (or z-drug) prescription		
Proportion of days supplied	Continuous	Over the 6-month interval
Any prescription of	Binary	Monthly
Mean dose dispensed	Continuous	Monthly
Antipsychotic prescription		
Proportion of days supplied	Continuous	Over the 6-month interval
Any prescription of	Binary	Monthly
Mean dose dispensed	Continuous	Monthly
Presence of 3+ CNS-active medications: antipsychotics, opioids, benzodiazepines, or antidepressants (including TCAs and trazodone)	Binary	Monthly
Statin—tracer outcome	Continuous	Over the 6-month interval
Proportion of days supplied		

RAI data will be used to identify dates of admission and discharge to define the appropriate set of residents contributing to each time period. We will also use RAI data to summarize relevant demographic and clinical characteristics of the cohort of nursing home residents cared for the participating physicians. We will use an open cohort design; all eligible residents present in the home at any observation time will be included in the analysis. To be eligible for analysis, the most responsible physician of the nursing home resident must have signed up to receive the report.

### Analysis

All primary analyses will be by intention-to-treat. The primary outcome (monthly number of CNS-active medications over 6 months pre- and post-intervention) will be analysed using a general linear mixed effects regression model; time will be specified as a continuous variable and a common secular trend will be imposed across all study arms with the effect of the intervention modelled as a slope deviation from the secular trend. The analysis will adjust for the size of each home as a fixed effect. Random intercepts and slopes for time will be specified for the unit of randomization (group of homes) and for the individual resident to account for cluster and individual autocorrelation. The primary comparison between the arms’ 6-month post-intervention will be estimated using least square mean differences, together with 95% confidence intervals. Because the traditional two-stage testing approach (i.e. an interaction test followed by dropping the interaction term if non-significant) can lead to bias in factorial trials [30], we will present both four-arm and factorial analyses.

We will examine whether the language in the key message in the report (i.e. the bolded statement declaring the number of additional/fewer patients are at risk from (or safe from risks of) benzodiazepines) is differentially associated with changes in prescribing. This is an uncontrolled element of the intervention, dependent on the observed distribution of performance of physicians relative to their peers. The relative performance of the physician compared to the comparator may act as an effect modifier because having additional patients at risk of harm or fewer patients safe from harm may invoke different responses than having fewer patients at risk or more patients safe. Additionally, it is plausible that recipients may respond more actively to simpler language (e.g. ‘7 additional residents in my practice may be at increased risk of harm’ may be easier to interpret than ‘7 fewer residents in my practice are safe from risk of harm’) because the simpler wording may make it easier for recipients to evaluate their performance relative to the comparator and respond accordingly. Thus, we will adjust for relative performance (i.e. better, same, or worse than comparator) and clarity of the statement (i.e. additional versus fewer) as a covariate and then conduct planned subgroup analyses to examine differential effects of the intervention across these subgroups (while acknowledging unmeasured confounding and uncertain power in these subgroup analyses).

We will also explore potential effect modification by baseline prescription rate, ownership status of nursing home, beds in the home, location (urban/rural), specialist utilization (proportion of patients with geriatrician or psychiatrist consultation within 6 months), plus primary physician characteristics (sex, years’ experience), as well as patient characteristics (age, sex, time at facility, levels of function and cognition). A planned sensitivity analysis will examine effects by extent of exposure to the intervention (i.e. fidelity of receipt, which assesses whether physicians downloaded their report).

The secondary outcomes, measured as proportion of days supplied with each prescription over the 6-month interval post-intervention, will be analysed using a general linear mixed effects regression with baseline proportion as a covariate. Random intercepts will be specified for the unit of randomization and for the individual resident. Monthly mean dose dispensed will be analysed as described for the primary outcome. Secondary outcomes measured as repeated binary proportions will be analysed as described for the primary outcome but using mixed effects logistic regression.

The RAI is needed to have accurate dates of admission and discharge from nursing homes, but linking data with the RAI can involve substantial delays. A planned interim analysis will be conducted using ODB data only to be used for HQO’s internal purposes.

### Power

In head-to-head trials where small changes to existing initiatives are tested and where each sequential change in the process of optimization is expected to offer cumulative benefit, it is necessary to power for small changes. In this pragmatic trial, the sample size is dependent on the number of providers who sign up for the reports. At the time of study launch, there will be physicians working in over 200 nursing homes who have signed up to receive the HQO Practice Reports. After grouping homes sharing providers together, we anticipate having approximately 160 clusters, with an average of 350 beds per cluster. In a 2 × 2 factorial design assuming no interaction and similar effects for each intervention, a test of each intervention at 6 months in an ANCOVA design will achieve 90% power to detect an absolute mean difference of 0.3 in the primary outcome (i.e. a reduction across arms in the mean number of CNS-active medications from 3 to 2.7). Based on previous data, we have assumed a standard deviation of 4, an intracluster correlation coefficient of 0.05, a cluster autocorrelation of 0.8, and an individual autocorrelation of 0.9 [31]. In the presence of an interaction effect, switching to a 4-arm comparison would require a slightly larger difference of 0.4 in the mean number of medications.

For our secondary outcome (any monthly prescription of antipsychotics), our sample size will achieve 90% power to detect an absolute difference of 1.2% (or a relative difference of 6%) from a reference arm proportion of 20% using six monthly pre- and post-intervention measurements, with an intracluster correlation coefficient of 0.05, a cluster autocorrelation of 0.8, and an individual autocorrelation of 0.9. In the presence of an interaction effect, switching to a 4-arm comparison would require a larger relative difference of 8.5%.

### Economic evaluation

We will conduct a cost-effectiveness analysis from the perspective of the publicly funded health care system. Total costs include costs associated with designing and delivering interventions and health care costs incurred by nursing home residents. We will use a micro-costing technique [32] to identify, measure, and value resources required for the interventions. HQO staff will provide the number and type of resources used for designing and delivering interventions. We will multiply the quantity of each type of resource required by its unit costs to obtain component-specific costs and overall costs for the interventions. Health care costs incurred by nursing home residents will be derived using the costing methodology developed at ICES [33].

We will estimate the incremental cost per unit change in monthly number of CNS-active medications as well as the incremental net benefit of the interventions using a general linear mixed effects regression model. A series of sensitivity analysis will be undertaken to examine the effect of conducting a complete case only analysis and of varying input parameters. Uncertainty in the analysis will be addressed by estimating 95% CIs using a non-parametric bootstrapping method. Results from the bootstrapping exercise will also be used to depict cost-effectiveness acceptability curves, which represent the probabilities that interventions being cost-effective over a range of potential threshold values that the health system may be willing to pay for an additional unit of effect.

#### Embedded theory-based process evaluation

The process evaluation involves a mixed-methods approach informed by the UK Medical Research Council’s Guidelines [34] to examine whether the intervention targets the intended constructs outlined in the program theory (Fig. 1) and understand the contextual factors that shape how the intervention works in practice.

To assess fidelity of receipt, we will examine the extent of exposure (i.e. timing and frequency of Practice Report downloads). We propose that exposure (fidelity of receipt) will be a key effect modifier of the intervention.

The intervention’s mechanism of action will be assessed via surveys of participating physicians across all four arms to measure theoretical constructs targeted by the intervention. Targeted constructs include intention, self-efficacy, outcome expectations, descriptive norms, and goal prioritization. To minimize response burden, a single validated question was included to measure each construct for the prescription of targeted medication classes (benzodiazepines, antipsychotics, and CNS-active medications), in addition to medications that the intervention does not directly target (e.g. statins). These will therefore provide the capacity to conduct a multiple-behaviour process evaluation [35] that will examine the presumed mechanisms underlying any changes observed in prescribing and whether these vary by experimental condition. Prospect theory suggests that when physicians make a decision, they consider risky (i.e. the decision to start/stop benzodiazepines), loss-framed messages may have a larger impact on their behaviour than gain-framed messages [19, 36]. At the same time, the importance of coherence between framing and how the recipient is oriented towards promoting gains or preventing harm is emphasized in Regulatory Fit Theory [37]. We assume that most physicians are focused on preventing harm when thinking about high-risk medications. We will include a questionnaire designed to assess whether individuals are promotion- or prevention-focused which has been validated in patient population [38]; we have adapted this tool to focus on healthcare professional behaviour. Responses will we be used to assess whether respondents’ regulatory focus moderates the effect of the feedback framing intervention.

To gain a more nuanced understanding of how and why the intervention worked as observed, we will conduct qualitative interviews with interested participants. We will examine whether participants view changes in prescribing as a risky behaviour, as assumed, or if they endorse generally positive experiences with prescribing these medications, and perceive their ongoing use as generally safe. We will also probe the underlying mechanisms through which the interventions may influence prescribing behaviour (Fig. 1). The constructs measured in the theory-based questionnaire will be represented within the interview topic guide. Interviews will focus on gaining additional insight into quantitative responses to the constructs assessed in the questionnaire while providing an opportunity for respondents to clarify any additional factors relevant to their experience of using the feedback report allocated to them.

### Recruitment and data collection

Working with HQO, we will send an invitation to all report recipients who download their report to complete a survey about the reports. We will follow an adapted Dillman protocol with a series of weekly reminders to optimize response rate [39]. Respondents will also be incentivized through a $25 raffle prize [40] provided by the research team (HQO, as a provincial agency, is prohibited from offering such incentives). Interview participants will be recruited using self-identification during the web-based surveys. Interested respondents will be contacted via e-mail to confirm interest and schedule a one-on-one, semi-structured telephone interview. An interview guide will focus on report use and ideas for improvement, prioritization of prescribing behaviour change in relation to the three prescribing indicators summarized in the report, and questions to probe the hypothesized mechanisms of action underlying the interventions (summarized in Fig. 1). It is expected interviews will last between 30 and 45 min. As interviews will focus on the experience of healthcare professionals in using feedback on aggregate data, there is very little risk of any patient-identifiable data being discussed. Nevertheless, all names and locations will be removed from transcripts to ensure that respondents are anonymous for the purposes of any reporting of results (presentations, manuscripts).

Items in the web-based survey used to measure constructs which are hypothesized antecedents of behaviour (Fig. 1) will be scored by participants using a 5-point Likert scale and described using mean and standard deviations (median and interquartile ranges in the case of skewed distributions). The survey also contains 11 items assessing regulatory focus, scored using a 9-point Likert scale, with high scores representing agreement and low scores disagreement. Six items assessed the extent of promotion focus, and five assessed the extent of prevention focus. We will assess the internal reliability of the items measuring each of the focus types (promotion/prevention) using Cronbach’s alpha. If internal consistency is <0.7, we will explore whether we can improve this by omitting individual items. We will then use the mean of the items measuring each focus type to create a composite score ranging from 1 to 9 for each focus type. The prevention focus composite score will then be subtracted from the promotion focus composite score. The resulting score will be used to classify the physicians as primarily prevention- or promotion-focused: physicians with positive scores will be classified as promotion-focused and physicians with negative scores as prevention-focused.

We will use multiple regression to explore the relationships between hypothesized predictor constructs (Fig. 1) and the dependent variable (intention to appropriately adjust prescribing) controlling for group allocation. We will then compare the post-intervention scores for the constructs across the intervention groups using independent samples *t* tests, conducting separate comparisons for each of the two main effects (message framing and professional comparator). We anticipate that approximately 250 providers will be allocated in the trial and that approximately 40% will download the Practice Reports. With an anticipated survey response rate of 40%, we expect to have 40 questionnaires available for analysis, evenly distributed between arms. With 20 survey responses in each group (presence or absence of each main intervention effect), we will have 80% power to detect a difference of 0.9 standard deviation units in any of the constructs between the two intervention arms using a two-sided *t* test at the 5% level of significance. We will use hierarchical mediation and moderation models to determine whether interventions work through hypothesized pathways to aid interpretations for generalization in various contexts. We will also conduct additional exploratory analyses to assess the impact of measurable provider, patient, and contextual characteristics on the outcomes.

The analytical method for the interviews will involve an iterative process of data collection and analysis using qualitative software NVIVO 10. Interviews will be analysed by two independent researchers using the framework method [41, 42]. This method of analysis involves six key steps: familiarization with the data, coding the data, indexing (applying the analytical framework), charting (summarizing the data from each transcript by category), and interpretation. A coding framework will be developed, with the key psychological constructs (Fig. 1) serving as overall themes. Open coding will be applied as required by the data to allow for the emergence of themes not captured within the constructs. The researchers will meet to discuss and refine the coding framework and themes. Throughout the indexing period, refinement will involve discussion with a third researcher and the wider study team as necessary, for example, to reach consensus on discrepancies and include the clinical perspective. At the charting stage, data will be re-arranged into tables and summarized according to the analytical framework. We will then re-examine the data to develop our interpretation of the mechanisms of effect of the interventions.

The framework approach ensures a systematic approach to summarizing and classifying the data, facilitating a comprehensive review of participant narratives while encouraging a higher-level conceptual analysis [42]. Using an interconnected analytic strategy allows for the constant refinement of themes while progressing towards the development of an overarching conceptual framework of how the intervention works and why. Recruitment will continue until saturation is reached; however, we anticipate conducting ~15–20 interviews based on similar studies [43]. We will employ a 10 + 3 rule, whereby at least 13 interviews will be conducted; if no additional themes are generated in the last three consecutive interviews, this will serve as evidence of data saturation. If not, interviews will continue until at least three consecutive interviews do not raise new themes. If less than 15 physicians consent to be contacted, we will contact them all for interview. Snowball sampling will be used to seek out contrasting cases, if possible.

#### Ethical considerations

This trial meets all ethical principles for a deviation from the general principles of research consent [44]. Specifically, the study intervention and data collection procedures pose no more than minimal risk for participating physicians and are unlikely to adversely affect their welfare. This is because these physicians already voluntarily signed up for the Practice Reports; HQO can design them in any reasonable fashion and evaluate the effects of their initiative so long as confidentiality is respected. Furthermore, the experiment would not be feasible without a waiver of consent [45]. Typical consent procedures would likely create selection bias (i.e. physicians likely to consent may systematically differ in the way they respond to framing). Therefore, the participating research ethics boards approved waiver of consent with provision of opt-out opportunities and a debrief at the time of outcome assessment (refer to Additional file 5).

## Discussion

This trial demonstrates how partnerships with health system organizations can create opportunities to advance implementation science [14]. We envision this trial as the first in a series of studies in our implementation science laboratory with HQO that test AF variations. The collaborative process described herein may offer lessons for others working in implementation science laboratories and illustrate how appropriate compromises can be reached in this context to achieve both scientific objectives and health system improvement goals.

Numerous components within AF may play a role in the effectiveness of the intervention; no single trial can answer all the remaining questions about how to optimize design and delivery. Further work will be needed both to optimize these aspects of AF and to test additional features. This is especially true because this trial is not without its limitations. The re-design process (see Additional file 1) was abridged to accommodate project deadlines. In addition, framing was difficult to operationalize in a clear manner because the data lacked clinical granularity; prescribing high-risk medications more often than the comparator may sometimes be appropriate, depending on the patient characteristics, and the feedback required carefully crafted content to acknowledge this issue. We cannot be certain that our elaboration of risk framing and benefit framing will be equally clear/understandable to recipients. Further, our hypothesis related to framing assumes that recipients of AF reliably perceive the decision to change (i.e. start or stop) benzodiazepine prescriptions to be a risky behaviour; this will be explored in the process evaluation. We also acknowledge that Regulatory Focus Theory [36] posits that the alignment between message delivery and the way in which a person perceives that task plays a role in how persuasive the message will be and its effect on performance. We emphasized preventing risks in the report because we felt this fit both the overall goal of the initiative (i.e. to limit high-risk prescribing) and the widely held medical dictum to ‘first do no harm’ [46]. We will assess participants’ regulatory focus in the process evaluation to help interpret the results. In general, other design aspects beyond those manipulated in this trial may create noise that drowns out any signal we may hope to find; we will explore the role of these aspects where possible to inform future research.

Learning about how physicians respond to positively versus negatively framed clinical performance feedback may have implications for a wider field of continuing professional education [47, 48]. This protocol demonstrates how one can manipulate theoretically informed components in a process that can both improve a specific intervention and provide generalizable insights. The general approach may be used to inform approaches to additional head-to-head trials and to generate new knowledge regarding how best to design and deliver implementation interventions.

### Trial status

The trial began December 2016, with 267 physicians from 160 clusters of nursing homes allocated to the four experimental conditions. The intervention period is ongoing.

## Additional files

Additional file 1: Details of the re-design of the intervention in preparation for this trial. (DOCX 28 kb)

Additional file 2: Hypothesized mechanism of action for each factor being manipulated in the trial. (DOCX 18 kb)

Additional file 3: Screenshot of the first page of the Practice Reports used prior to this trial. (DOCX 167 kb)

Additional file 4: Screenshots of the first page of the four versions of the Practice Reports tested in this trial. (DOCX 504 kb)

Additional file 5: Waiver of consent documents: letter of information and debrief to participants in the trial. (DOCX 28 kb)

## Abbreviations

AF: Audit and feedback
ANCOVA: Analysis of covariance
APM: Antipsychotic medication
BCT: Behaviour change technique
BZD: Benzodiazepine
CCRS: Continuing Care Reporting System
CI: Confidence interval
CIHI: Canadian Institute for Health Information
CNS: Central nervous system
CPSO: College of Physician and Surgeons of Ontario
HQO: Health Quality Ontario
ICES: Institute for Clinical Evaluative Sciences
IQR: Interquartile range
ODB: Ontario drug benefits
OHIP: Ontario health insurance program
RAI: Resident Assessment Instrument
RPDB: Registered persons database
TCA: Tricyclic antidepressants
