# Supplementary material for: Testing feedback message framing and comparators to address prescribing of high-risk medications in nursing homes: protocol for a pragmatic, factorial, cluster-randomized trial
Source: Implement Sci. 2017 Jul 14;12:86. doi: 10.1186/s13012-017-0615-7 (PMC5512954; doi:10.1186/s13012-017-0615-7)
Supplement: Supplementary file 1 — Details of the re-design of the intervention in preparation for this trial. (DOCX 28 kb) [file 13012_2017_615_MOESM1_ESM.docx]

**Additional file 1 - Details of Redesign of HQO Practice Reports in preparation for this study**

The HQO reports are generated by a multidisciplinary team, including clinicians, epidemiologists, and quality improvement experts, supported by input from front-line clinicians, health services researchers, sector organizations and associations, and policy makers. HQO relies on best evidence, advice from the groups listed above, and established methods (e.g., modified Delphi process) to select and develop indicators and generate suggested action plans to support quality improvement for the Report.

The team of implementation scientists and experienced practitioners at HQO worked in partnership to develop this trial. The decision to focus on the Long-Term Care Practice Reports for this initial trial was collaborative; the choice of indicators to include in the practice profiles was driven by the standard process that HQO employs to select and develop indicators; decisions regarding research methods were predominantly led by the scientists; and the design of the reports (i.e., how the messages in the report conveyed the results for each indicator) was collaborative, as described below.

Since October 2015, HQO has produced confidential, individual practice reports approximately every three months for physicians working in nursing homes in Ontario. The feedback reports describe aggregated prescribing rates for selected medications prescribed for patients cared for by the physician-recipient. The reports also include information regarding the clinical and demographic features of recipients’ nursing home residents. To protect confidentiality, data are suppressed and described as ‘not reportable’ for any cell sizes from one to five. See Supplementary File 3 for screenshot of the first page of the HQO Long-Term Care Practice Reports used prior to this study.

A joint committee led by Ontario Medical Association and Ontario’s Ministry of Health and Long-Term Care provided guidance on topic areas to be included in the report. Initially, the reports focused on antipsychotic prescribing as they are known to have substantial risks in the elderly, to be relatively overprescribed given the known risk/benefit ratio, and appear to be amenable to quality improvement interventions in the nursing home setting [1-3]. (See *Additional File 3* for an example of the feedback reports produced by HQO prior to this study.) Concurrent with the launch of this study, new indicators were added to the reports as HQO was guided to include medications associated with the increased risk of falls [4-6]. Thus, in addition to antipsychotics two additional high-risk medication indicators were added: benzodiazepine prescriptions and a metric showing the proportion of patients receiving three or more central nervous system (CNS) active medications (i.e., patients receiving 3 or more medications from the following classes: antipsychotics, benzodiazepines, opioids, and antidepressants). HQO decided to include this indicator because it wanted to prompt physicians working in nursing homes to consider ways to limit cumulative burden of these medication classes for patients in terms of cognition and other side effects related to an increased risk of falls [7].

The addition of new indicators presented an opportunity to consider options for how to design the report. To that end, we conducted heuristic evaluations supported by an expert in human factors and interface design (HOW) and used think-aloud methods [8] in remote usability tests with a total of 8 potential users (physicians previously naïve to the reports who work in nursing homes) across 2 testing cycles. Together, research team design critiques, consultation with HQO and its advisors, and user testing led to 21 iterative versions of the report, some representing major changes and others, minor revisions. The final revised report addressed user concerns with a report design that a) used text and data-visualization to place greater emphasis on the gap between desired and actual care, b) openly acknowledged limitations in the data in terms of assessing individual-level benefits or harms, and c) focused on the potential for patient benefit if report recipients re-examined their high-risk prescribing practices, with a focus on benzodiazepines, as many users felt ready for a new focus beyond antipsychotics and some felt they had no further capacity to improve antipsychotic prescribing.

See *Additional file 4* for an example of the feedback reports included in this study. The redesigned reports provide feedback in three ways: 1) visually, using line graphs with quarterly historical data on the practice and the comparator for two years up to the current date to show progress over time, 2) numerically, as a summary percentage of their practice and the comparator for the most recent quarter to demonstrate current performance, and 3) narratively, as a statement expressing the gap between comparator and actual performance. For the latter aspect, we deliberately expressed the gap between the comparator and actual performance in a bolded, declarative statement as the number of additional/fewer nursing home residents receiving benzodiazepines because it may be easier for physicians to conceptualize discrete individuals than percentages [9].

1. Rochon PA, Stukel TA, Sykora K, Gill S, Garfinkel S, Anderson GM, Normand SL, Mamdani M, Lee PE, Li P, Bronskill SE, Marras C, Gurwitz JH: **Atypical antipsychotics and parkinsonism.** Arch Intern Med 2005, 165:1882-8.
2. Schneider LS1, Dagerman KS, Insel P. **Risk of death with atypical antipsychotic drug treatment for dementia: meta-analysis of randomized placebo-controlled trials.** JAMA 2005, 294:1934-43.
3. Avorn J, Soumerai SB, Everitt DE, Ross-Degnan D, Beers MH, Sherman D, Salem-Schatz SR, Fields D: **A randomized trial of a program to reduce the use of psychoactive drugs in nursing homes.** N Engl J Med 1992, 327:168-73

4. Leipzig RM, Cumming RG, Tinetti ME: **Drugs and falls in older people: a systematic review and meta-analysis: II. Cardiac and analgesic drugs.** J Am Geriatr Soc 1999, 47:40-50.

5. Leipzig RM, Cumming RG, Tinetti ME: **Drugs and falls in older people: a systematic review and meta-analysis: I. Psychotropic drugs.** J Am Geriatr Soc 1999, 47:30-9.

6. Ray WA, Thapa PB, Gideon P: **Benzodiazepines and the risk of falls in nursing home residents.** J Am Geriatr Soc 2000, 48:682-5.

7. American Geriatrics Society Beers Criteria Update Expert Panel: **American Geriatrics Society 2015 Updated Beers Criteria for Potentially Inappropriate Medication Use in Older Adults.** *J Am Geriatr Soc* 2015, **63:**2227-2246.

8. Li AC, Kannry JL, Kushniruk A, Chrimes D, McGinn TG, Edonyabo D, Mann DM: **Integrating usability testing and think-aloud protocol analysis with "near-live" clinical simulations in evaluating clinical decision support.** Int J Med Inform 2012, 81:761-72.

9. Gigerenzer G, Gaissmaier W, Kurz-Milcke E, Schwartz LM, Woloshin S: **Helping Doctors and Patients Make Sense of Health Statistics.** *Psychol Sci Public Interest* 2007, **8:**53-96.
