# Supplementary material for: Testing feedback message framing and comparators to address prescribing of high-risk medications in nursing homes: protocol for a pragmatic, factorial, cluster-randomized trial
Source: Implement Sci. 2017 Jul 14;12:86. doi: 10.1186/s13012-017-0615-7 (PMC5512954; doi:10.1186/s13012-017-0615-7)
Supplement: Supplementary file 2 — Hypothesized mechanism of action for each factor being manipulated in the trial. (DOCX 18 kb) [file 13012_2017_615_MOESM2_ESM.docx]

**Additional file 2 - Hypothesized Mechanism of Action of the factors manipulated in this experiment**

*Factor 1- Comparator*

Consistent with Goal Setting Theory [1, 2] and Social Cognitive Theory of Self-Regulation [3], we hypothesize that providing feedback on performance in relation to the top quartile of performers will provide a social comparison and set a self-standard for a difficult but achievable goal which will lead to reduced high-risk medication prescribing, relative to social comparison to a less challenging social reference. Consistent with the above-mentioned theories, we further hypothesize that this effect will operate on prescribing behaviour by increasing awareness of social standards (descriptive norms), increased self-efficacy, and increased motivation.

*Factor 2- Framing*

We hypothesize that feedback framed to emphasize the number of patients at risk of harm will be more effective than feedback emphasizing the number of patients safe from risk of harm. We assume that risk-framed messaging regarding prescription of these high-risk medications will tend to increase the likelihood of a behaviour change by increasing physicians’ outcome expectations regarding potential harms, thereby increasing priority and motivation to reduce prescribing. Consistent with previous research, we further hypothesize that risk-framed messages will be more persuasive when individuals have high self-efficacy, since a lack of confidence may lead risk-framed messages to invoke a greater sense of threat [4, 5]. Thus, we will explore whether any effect of message-framing on prescribing is moderated by self-efficacy.

1. Locke EA, Latham GP: **Building a practically useful theory of goal setting and task motivation. A 35-year odyssey.** *Am Psychol* 2002, **57:**705-717.

2. Locke EA, Latham GP: *A theory of goal setting & task performance.* Englewood Cliffs, N.J.: Prentice Hall; 1990.

3. Bandura A: **Social cognitive theory of self-regulation.** *Organizational Behavior and Human Decision Processes* 1991, **50:**248-287.

4. Covey J: **The role of dispositional factors in moderating message framing effects.** *Health Psychol* 2014, **33:**52-65.

5. Riet Jvt, Ruiter RAC, Werrij MQ, de Vries H: **The influence of self-efficacy on the effects of framed health messages.** *European Journal of Social Psychology* 2008, **38:**800-809.
