# Supplementary figures and images for: Testing feedback message framing and comparators to address prescribing of high-risk medications in nursing homes: protocol for a pragmatic, factorial, cluster-randomized trial
Source: Implement Sci. 2017 Jul 14;12:86. doi: 10.1186/s13012-017-0615-7 (PMC5512954; doi:10.1186/s13012-017-0615-7)

**Additional file 3 - Screenshot of the first page of the Practice Reports used prior to this trial**

**­­**


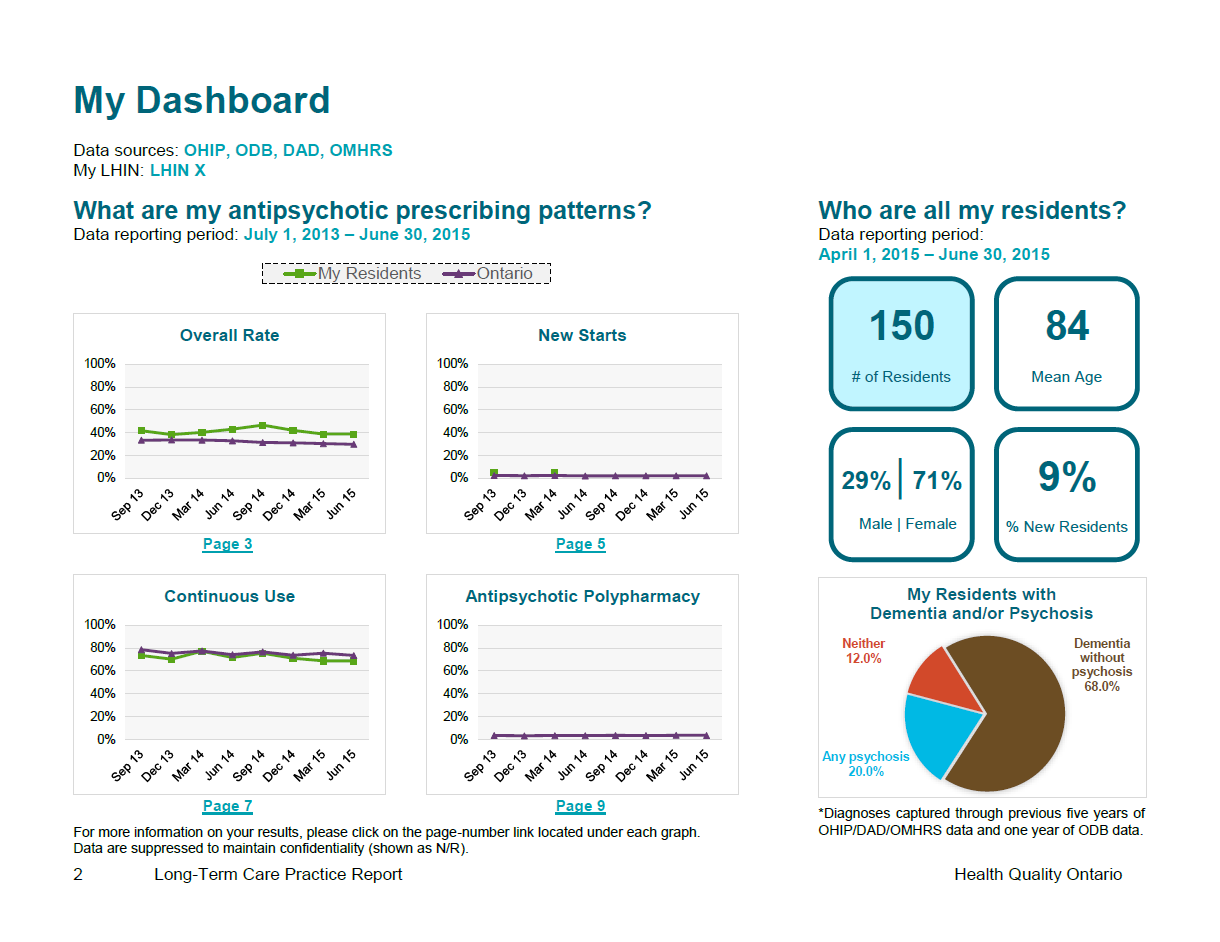

Supplement: Supplementary file 3 — Screenshot of the first page of the Practice Reports used prior to this trial. (DOCX 167 kb) [file 13012_2017_615_MOESM3_ESM.docx]
