# Supplementary material for: Testing feedback message framing and comparators to address prescribing of high-risk medications in nursing homes: protocol for a pragmatic, factorial, cluster-randomized trial
Source: Implement Sci. 2017 Jul 14;12:86. doi: 10.1186/s13012-017-0615-7 (PMC5512954; doi:10.1186/s13012-017-0615-7)

**Additional file 4 - Screenshots of the first page of the four versions of the Practice Reports tested in this trial**


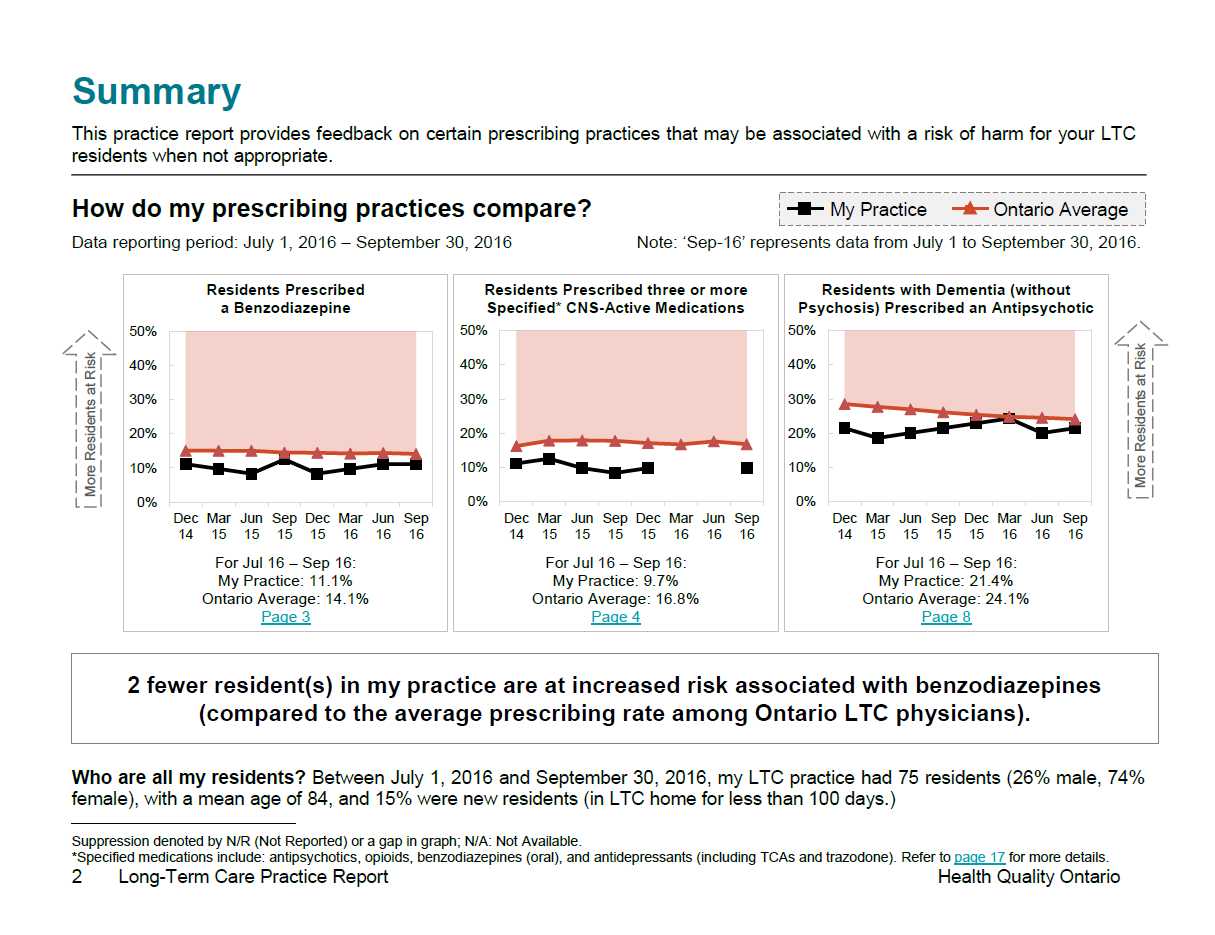


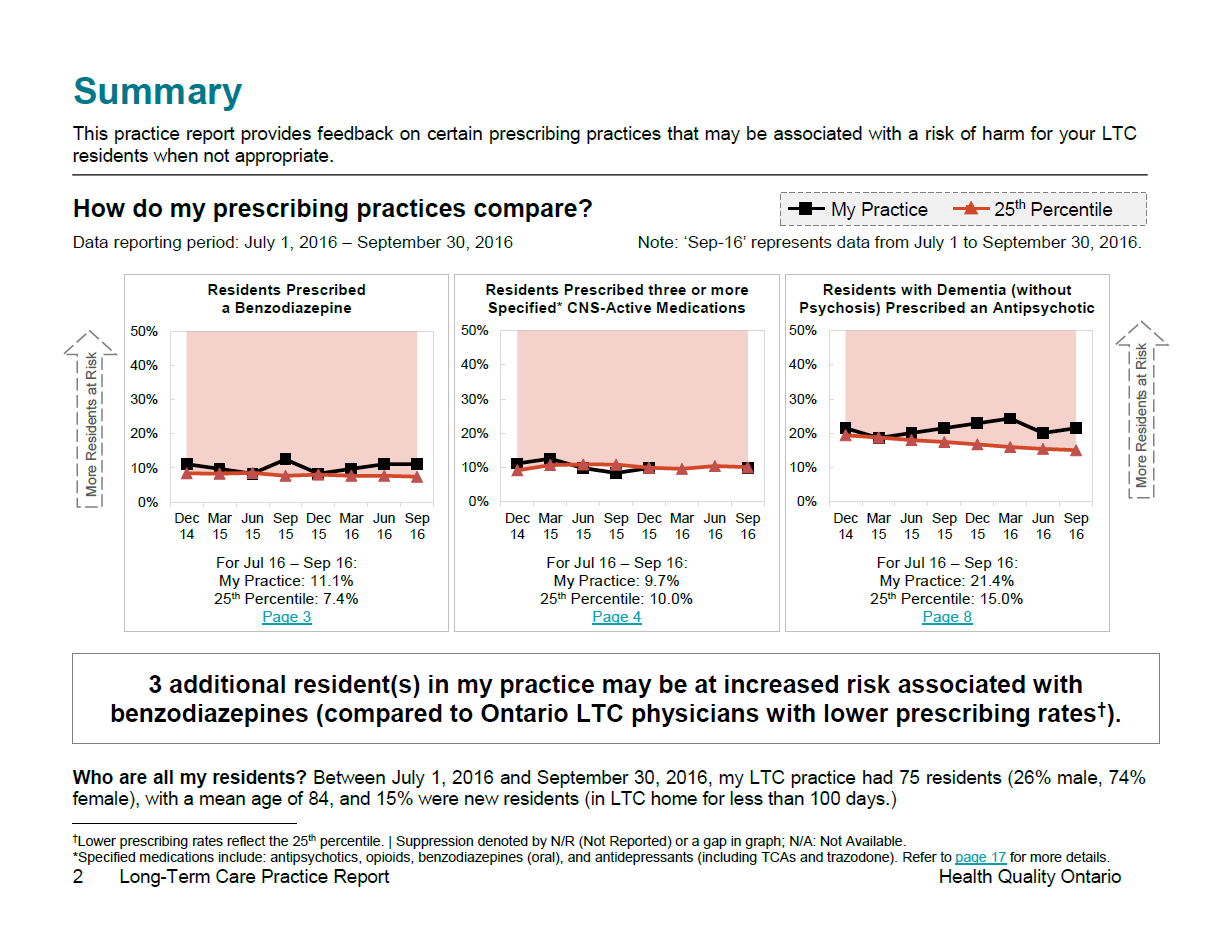


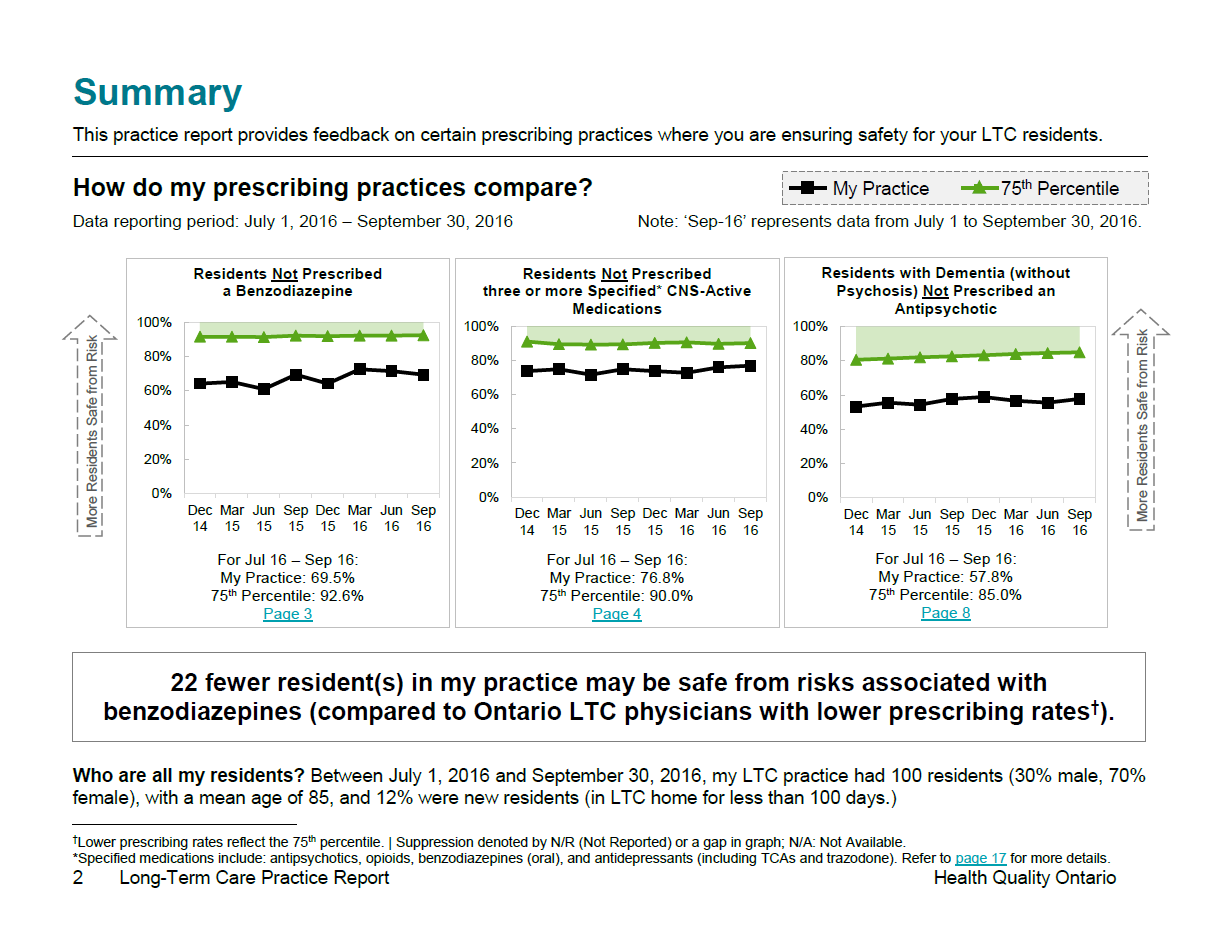


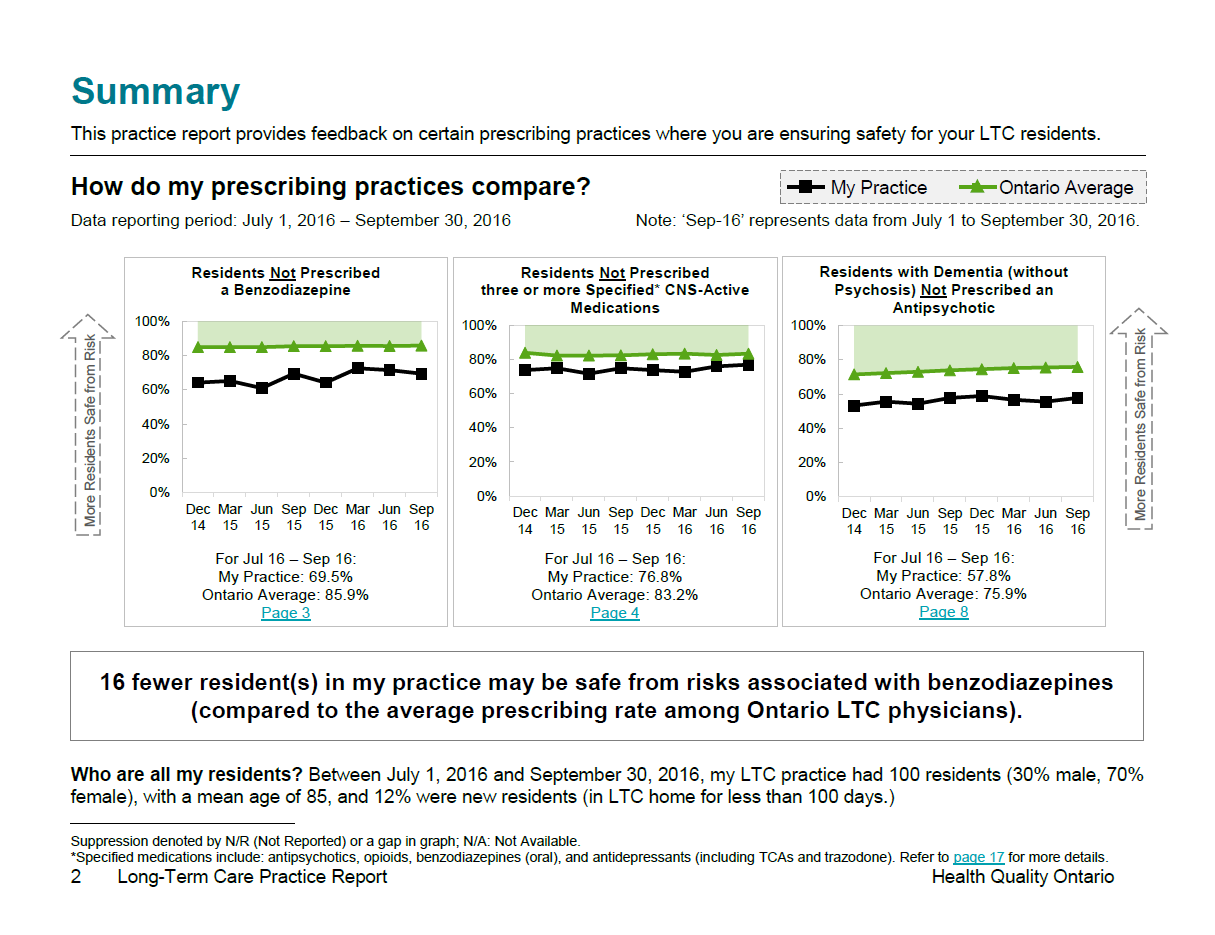

Supplement: Supplementary file 4 — Screenshots of the first page of the four versions of the Practice Reports tested in this trial. (DOCX 504 kb) [file 13012_2017_615_MOESM4_ESM.docx]
