# Supplementary material for: Testing feedback message framing and comparators to address prescribing of high-risk medications in nursing homes: protocol for a pragmatic, factorial, cluster-randomized trial
Source: Implement Sci. 2017 Jul 14;12:86. doi: 10.1186/s13012-017-0615-7 (PMC5512954; doi:10.1186/s13012-017-0615-7)
Supplement: Supplementary file 5 — Waiver of consent documents: letter of information and debrief to participants in the trial. (DOCX 28 kb) [file 13012_2017_615_MOESM5_ESM.docx]

**Additional file 5 - Waiver of consent documents**

**Letter of information upon sign-in to Practice Reports**

**Letter of information: Optimizing HQO Practice Profiles**

Health Quality Ontario (HQO) is committed to continuously improving the Practice Reports to make them as useful as possible for you. Therefore, you may notice changes in the way your Practice Report looks. This is because we are testing different ways of presenting information to understand whether some ways work better than others. We will regularly update you about the findings and how it helps us improve the Practice Reports we provide you.

If you wish further information about our efforts to optimize the Reports or have feedback for us about the reports, please contact us at [xxx@hqontario.ca](mailto:xxx@hqontario.ca).

**Example of debrief notice** **for participating physicians**

**Debrief: Optimizing HQO Practice Profiles**

Health Quality Ontario (HQO) is committed to continuously improving the Practice Reports to make them as useful as possible for you. Over the last six months we have found that the Reports result in greater improvements when they include [achievable benchmarks] rather than [the provincial average score]. We also found that framing the quality indicators in a [positive] light results in greater improvement. Therefore, we will be incorporating these design features into our standard approach. We believe we can continue to find ways to optimize the Reports; we will regularly update you about our efforts to improve the Practice Reports we provide you.

If you wish further information about our efforts to optimize the Reports or have feedback for us about the reports, please contact us at [xxx@hqontario.ca](mailto:xxx@hqontario.ca). If you wish to have your anonymized data removed from the analysis comparing different types of feedback reports, or if you wish to opt-out of similar evaluations of Practice Reports in the future, please contact us at [xxx@hqontario.ca](mailto:xxx@hqontario.ca).
